# Opposing Changes in Cerebellar Dopaminergic Genes Co-Expression Networks in Different Models of Neurodevelopmental Disorders

**DOI:** 10.3390/ijms27125508

**Published:** 2026-06-18

**Authors:** Anastasia D. Belskaya, Zoia S. Fesenko, Anna B. Volnova, Raul R. Gainetdinov, Anastasia N. Vaganova

**Affiliations:** 1 Institute of Translational Biomedicine, St. Petersburg State University, Universitetskaya Nab. 7/9, 199034 St. Petersburg, Russia; 2 Center for Transgenesis and Genome Editing, St. Petersburg State University, Universitetskaya Nab. 7/9, 199034 St. Petersburg, Russia

**Keywords:** cerebellum, transcriptome, RNA-seq, neurodevelopmental disorders, ASD, autistic spectrum disorders, dopamine

## Abstract

While the cerebellar dopaminergic system is suggested to be implicated in neurodevelopmental disorders, especially autism spectrum disorder (ASD), the details of its disturbances remain unclear. We performed a comparative analysis of human (GTEx) and mouse (GSE144046, GSE144277) transcriptomes, complemented by RT-qPCR in DAT-KO rats, to identify dopaminergic gene associations in the normal cerebellum and neurodevelopmental disorder models. Pairwise dopaminergic gene correlations were generally weak, with a slight increase in interaction complexity in ASD models. However, weighted gene co-expression network analysis identified a robust gene module involving *Comt*, which was consistently associated with synaptic translation across mouse datasets. These associations reflect regulatory processes in the whole cerebellum, which is commonly represented in rodent studies but absent in human data, which are acquired in studies of cerebellar subregions. ASD modeling exerted contrasting effects: *Cul3* haploinsufficiency increased the number of genes involved in the module with a decrease in connectivity, while *Mbd5* haploinsufficiency led to module collapse. These findings confirm neurodevelopmental disorders as a heterogeneous condition where divergent backgrounds uniquely rewire cerebellar dopaminergic networks. Considering the cerebellum’s role in ASD and that some ASD medications target the dopamine system, further investigation of these identified trends may support the development of more personalized therapeutic approaches.

## 1. Introduction

Despite its relatively compact size, the cerebellum contains nearly 80% of all brain neurons, which show a wide range of morphofunctional diversity. This population includes inhibitory stellate, basket, Purkinje (PCs), and Golgi cells, alongside excitatory granule and unipolar brush cells, and various glial types [1,2]. While the primary cerebellar output is mediated by inhibitory PCs projecting via deep cerebellar nuclei to the thalamus and back to the cortex, forming the classic circuit for motor coordination and error correction [3,4]. However, complex cerebellar function also involves less-studied pathways, such as projections to the substantia nigra, which elevate striatal dopamine levels, increase locomotion, and encode reward values [5], as well as connections to the striatum and prefrontal cortex via the thalamus and monosynaptic inputs to ventral tegmental area (VTA) dopaminergic neurons [6,7].

The cerebellum coordinates and controls behavior by constructing internal models that compare novel sensory input with predictions [8,9]. Its organization is topographically distinct: the anterior cerebellum is reciprocally connected to sensorimotor cerebral cortices, mediating motor behaviors, whereas the posterolateral cerebellum interfaces with non-motor association areas to support cognitive functions [4,9]. Additionally, the cerebellum contributes to social and affective processing, including the recognition of emotional stimuli, such as faces or voices, reward processing, mentalizing others’ emotional states, and the awareness of subjective feelings [4,10,11].

The cerebellum interacts with the dopaminergic system in three distinct ways, including endogenous dopamine production, reception of extrinsic dopaminergic input (primarily from the ventral tegmental area [10]), and regulatory control of dopaminergic circuits in the midbrain, striatum, and neocortex [12,13,14,15,16]. The main endogenous source of dopamine in the cerebellum are PCs. Tyrosine hydroxylase (TH) and dopamine transporter (DAT) have been identified within these cells, particularly in the posterior cerebellum. Additionally, PCs may synthesize dopamine via cytochrome P450 [15]. TH insufficiency in these cells correlates with impaired behavioral flexibility [6,17]. Large non-traditional neurons (including Landau neurons), which participate in corticonuclear and corticocerebellar projective circuits, also express DAT [17,18]. Widespread expression of all five dopamine receptor subtypes (DRD1–DRD5) has been demonstrated in rodents and humans across stellate, basket, granule, and PCs [10,17,19]. DRD1 activation inhibits PCs’ activity, which results in a reduction in locomotor activity and social interaction [15]. Meanwhile, DRD1 inhibition in deep cerebellar nuclei impairs spatial navigation, social recognition, error prediction, and working memory [17,20]. DRD2 is preferentially expressed in the postsynaptic compartments of the PC layer; its activation similarly decreases PC excitation and hinders sociability [10].

The cerebellum follows a complex developmental trajectory that is highly susceptible to insults, such as premature birth or genetic predispositions, which can trigger clinical symptoms of both disorders [21,22,23,24,25]. Mature cerebellar damage also affects social interactions [1,9,26,27,28,29,30]. Notably, the sensorimotor cerebellar regions mature earlier than the posterolateral regions associated with cognitive and social functions [23,24]. Disruption of sensory feedback processing in ASD and ADHD may contribute to motor differences in patients [16,29,30]. Simultaneously, disruptions in cerebellar connectivity with cortical areas involved in executive function and language (e.g., prefrontal cortex, posterior parietal cortex, and superior temporal gyrus) likely underpin the deficits seen in ASD and ADHD [9,21,31,32,33,34,35,36]. In ASD specifically, sensory over-responsivity correlates with increased connectivity to motor regions and weakened links to socio-emotional areas, particularly the prefrontal cortex [21,29,31].

Morphological studies of the autistic cerebellum reveal reduced volume, neurotransmitter imbalances, aberrant glial activation, and monocyte accumulation [8,37,38,39]. Furthermore, individuals with ASD consistently exhibit reductions in the number, size, and dendritic arborization of PCs, alongside disrupted vesicular trafficking [27,31,40,41,42]. While ADHD is also associated with reduced cerebellar volume and altered neurochemistry, these two disorders impact different regions; ADHD is characterized by impairments in attention networks and voluntary movement control [16,18,24,43,44]. Although the role of cerebellar dopamine in these pathologies requires further study, low DRD2/3 availability in the cerebellum has already been correlated with social-communication difficulties in ASD [45].

The dopamine transporter knockout (DAT-KO) rats are one of the models of disrupted dopaminergic neurotransmission-related disorders [46]. These knockout animals are characterized by an eightfold increase in extracellular dopamine concentration within the striatum and a distinct phenotype compared to wild-type rats, including dwarfism, hyperactivity, stereotypy, and behavioral rigidity [46,47,48,49,50]. Heterozygous rats DAT-Het, which carry only one mutant allele and exhibit less pronounced physiological differences from wild-type rats, nonetheless demonstrate impaired social behavior [51]. Consequently, the complete or partial absence of DAT makes these rats an excellent model of persistent hyperdopaminergia for investigating the mechanisms of dopamine-associated disorders such as ADHD or ASD [50,52]. Consequently, due to the lack of DAT, dopamine levels in the DAT-KO rats’ dopamine synapse clefts are increased [46]. However, tissue dopamine concentrations are decreased in the midbrain, striatum, medulla oblongata, and spinal cord of these animals but increased in the prefrontal cortex and hippocampus and remain stable in the cerebellum [53]. Additionally, previous studies have demonstrated that DAT-KO rats manifest volume loss in the dorsal striatum, which correlates negatively with an increase in cerebellar volume [54]. So the dopamine system’s dysfunction seems to impact the cerebellum, despite the stable dopamine level in this structure.

The aim of the present study is to characterize the expression patterns of genes involved in dopamine signaling and metabolism in normotypically developed cerebellums and compare these findings with relevant animal models of neurodevelopmental disorders like ASD or ADHD, applying public transcriptomic data and DAT-KO rets cerebellar samples. In particular, we analyzed genes whose products are involved in dopamine synthesis (*TH*, *DDC*), storage (*SLC6A3*, *SLC18A2*), signaling (*DRD1–5*), and utilization (*COMT*, *MAOA*, and *MAOB*).

## 2. Results

### 2.1. Dopamine System’s Gene Expression Pattern in Cerebellum in GTEx Data

Two datasets for the human cerebellum are available in the Gene-Tissue Expression GTEx database, including the datasets representing RNA-seq-generated expression profiles of the left cerebellar hemisphere and vermis. Taking into account non-homogeneous cerebellar molecular breakdown, as well as functional and evolutionary differences in these cerebellar substructures, we analyzed these datasets separately.

**Figure 1:**
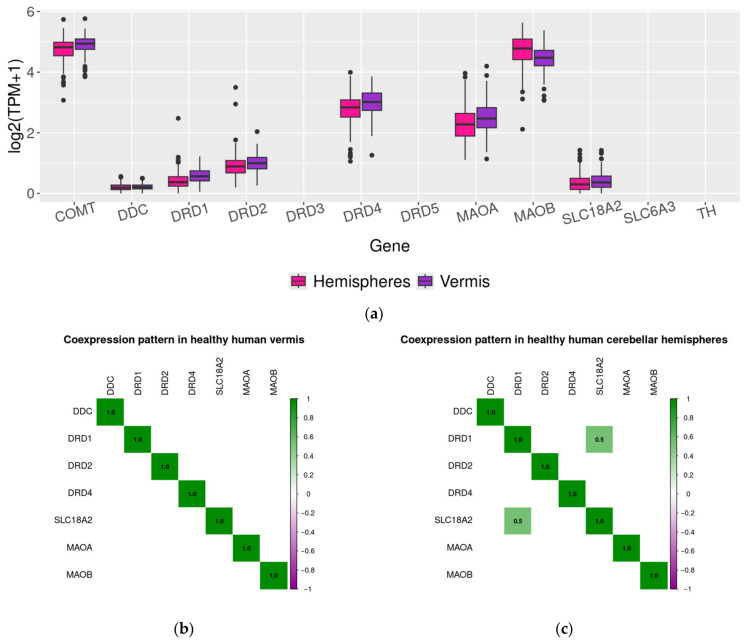
Dopaminergic gene expression and co-expression patterns in GTEx human cerebellum RNA-seq data. (**a**) mRNA expression profiles of the investigated genes. (**b**,**c**) Correlation matrices for the expression of the investigated genes in the vermis and cerebellar hemispheres. The values indicate Spearman’s correlation coefficients with statistical significance set at *p* < 0.05. TPM—transcripts per million.

The expression levels of dopaminergic genes across the two datasets were generally congruent. *TH*, *SLC6A3*, *DRD3*, and *DRD5* expression was not detected in both the left cerebellar hemisphere and the vermis, whereas *DDC*, *DRD1*, *DRD2*, *DRD4*, *MAOA*, and *SLC18A2* reached moderate levels of approximately 10 transcripts per million (TPM) or lower in both regions. The most expressed genes were *COMT* and *MAOB* (Figure 1a).

Due to the significant methodological discrepancies in tissue collection and preservation (PAXgene fixation vs. fresh frozen samples) between two datasets, we opted not to perform a formal statistical comparison or differentially expressed genes (DEG) tests between these two groups. Such a comparison could lead to misleading interpretations, as observed variances may stem from technical artifacts rather than biological divergence.

No significant correlations were identified between expression levels of dopaminergic genes’ mRNAs, both in hemisphere samples and the vermis, with the exception of a weak correlation between *DRD1* and *SLC18A2* mRNA expression (*ρ* = 0.5, *p* < 0.05, Figure 1b,c).

### 2.2. MAOB Gene mRNA Is Co-Expressed with Microglial Genes in Human Cerebellar Samples

**Figure 2:**
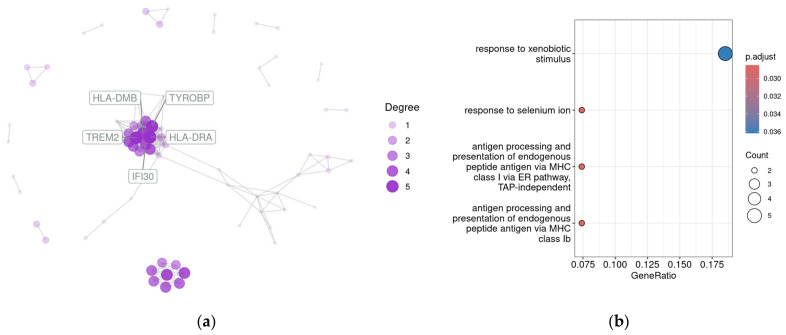
Co-expression network involving *MAOB* gene in “Brain—Cerebellar hemispheres” GTEx dataset: (**a**) general network topology, hub genes identified, (**b**) e Dotplot presenting the enrichment of Gene Ontology Biological Process (GO BP) terms for the cluster of genes co-expressed with MAOB mRNA. The *y*-axis displays the enriched GO terms, while the *x*-axis represents the Gene Ratio. The size of the dots corresponds to the count of genes enriched in each pathway, and the color intensity indicates the statistical significance (adjusted *p*-value).

To evaluate possible functional associations of dopaminergic genes in healthy cerebellum, we performed weighted gene co-expression network analysis (WGCNA). Integration into the co-expression network was observed only for *MAOB* mRNA in the hemisphere samples. This network exhibits a sparse topology, characterized by low connectivity and weak inter-node correlations. The identified network hubs are primarily involved in antigen presentation and myeloid-related functions (*CD68*, *IFI30*, *HLA-DRA*, *HL-DMB*, *TYROBP*, *TREM2*), including microglial activity (Figure 2a).

The genes co-expressed with *MAOB* in this network show minimal connectivity with one another and are weakly integrated into the overall network topology (refer to Supplementary Data S1 for visualization). Among the genes in this module, five are involved in the response to xenobiotics and two are associated with antigen presentation; the remaining genes appear to lack shared functional pathways (Figure 2b).

### 2.3. GEO Data Search and Inclusion

**Figure 3:**
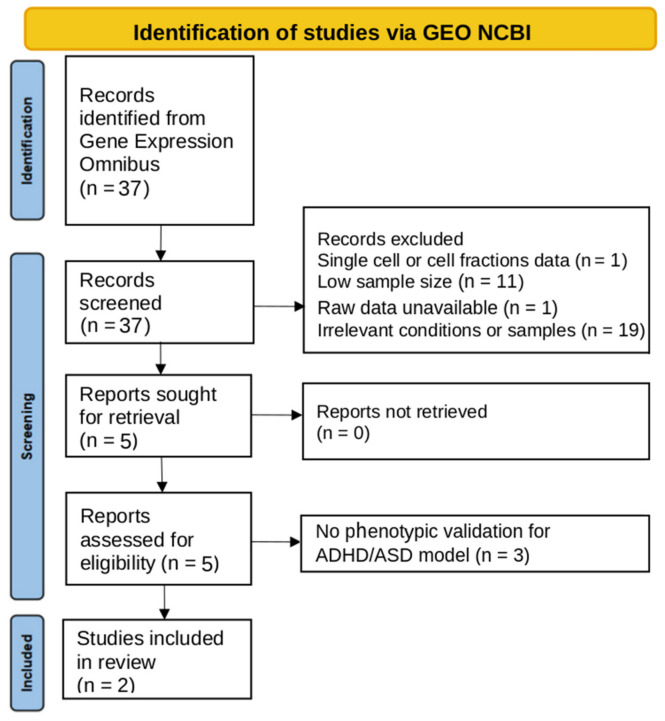
Flowchart of the data search.

Following a preliminary screening of the Gene Expression Omnibus (GEO) database, we successfully identified 37 relevant records. After a rigorous filtering process to remove non-relevant or incomplete datasets, we narrowed the selection down to five primary reports for final identification. Finally, we selected two specific RNA-seq datasets, which are explicitly listed in Table 1, for inclusion into our comparative analysis to ensure robust results. Three datasets were excluded: these data were generated in studies devoted to “molecular” modeling, in which consequences of intervention were examined by molecular biology approaches but were not validated by behavior tests, neither in the paper nor in references. The GEO data search is summarized in Figure 3.

### 2.4. Distribution of Dopaminergic Genes mRNA in GEO Cerebellar Datasets

**Figure 4:**
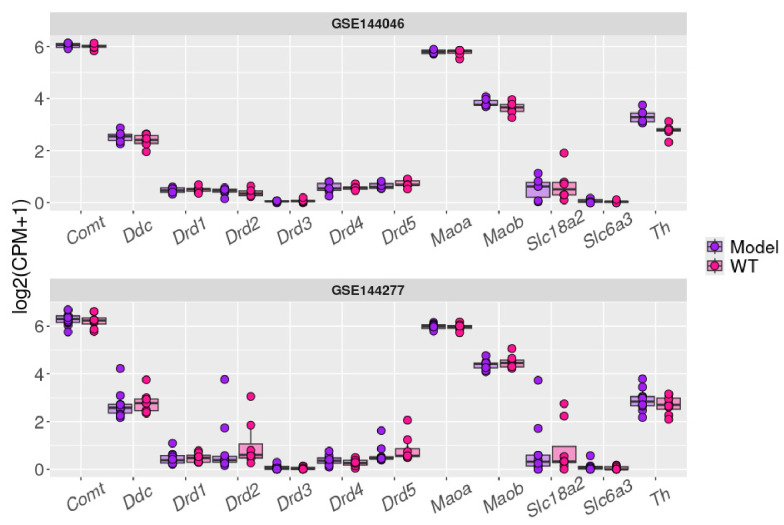
Dopaminergic genes expression in mouse cerebellum samples represented in GEO144046 and GSE144277 GEO datasets. CPM—counts per million.

In contrast to the GTEx results, all analyzed genes were detected in mouse cerebellum samples from the GSE144046 and GSE144277 datasets (Figure 4).

Expression profiles were congruent across datasets and showed no significant alterations in ASD mouse models compared to WT littermates (refer to Supplementary Data S2 and S3 for DEG analysis details).

**Figure 5:**
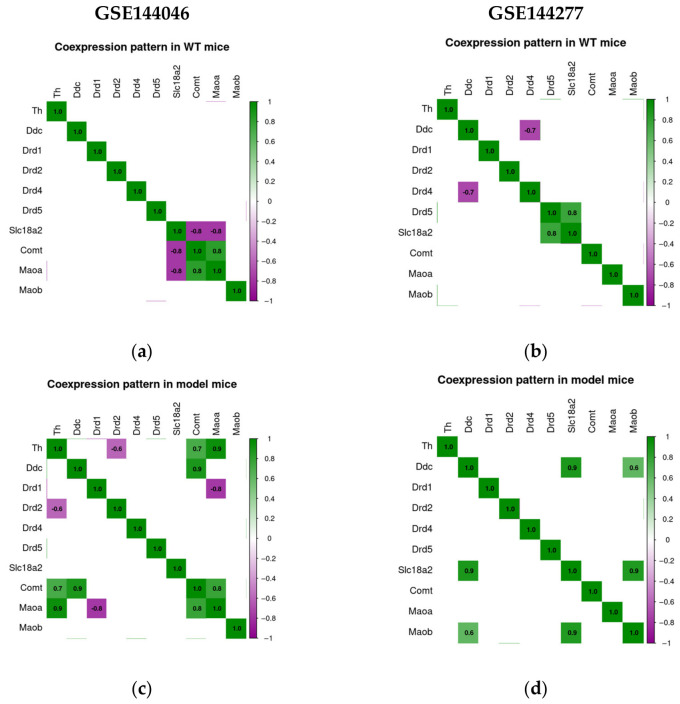
Co-expression networks of dopaminergic genes in the mouse cerebellum. (**a**,**b**) Co-expression of dopaminergic genes in the cerebellum in wild-type (WT) mice from the GSE144046 and GSE144277 datasets. (**c**,**d**) Co-expression of dopaminergic genes in the cerebellum in *Mbd5* and *Cul3* heterozygous knockout models from the respective datasets. The values indicate Spearman’s correlation coefficients with statistical significance set at *p* < 0.05.

However, the co-expression patterns of dopaminergic genes were not congruent between the two datasets, despite the mice being age-matched (P35 in GSE144046 and 8 weeks/P56 in GSE144277). These discrepancies likely arise from differences in sampling, pre-analytical processing, or analytical techniques. Notably, *Slc18a2* exhibited contrasting co-expression relationships in WT mice across the datasets: it was negatively correlated with *Comt* and *Maoa* in GSE144046 but showed positive co-expression in GSE144277 (Figure 5a,b).

Co-expression profiles in the ASD models were also not consistent. The only shared feature was an increase in the complexity of dopaminergic gene relationships in ASD models compared to WT littermates. Interestingly, in both models, *Ddc* became integrated into the network, with its expression correlating with genes involved in dopamine degradation (i.e., *Comt* and *Maoa* in GSE144046 and *Maob* in GSE144277). Similarly, an association between the *Th* and *Comt* genes was revealed in the GSE144046 dataset (Figure 5c,d).

### 2.5. Comt Participates in Weighted Gene Co-Expression Networks Enriched with Genes of Transcription/Transaltion Apparatus

To evaluate the involvement of dopaminergic genes in the co-expression network, we performed WGCNA (refer to Supplementary Data S2 and S3 for GSE144046 and GSE144277, respectively). We found that only *Comt* consistently passed the filtration criteria after selecting the top 5000 most variable genes in both datasets. Additionally, in the GSE144046 dataset, the *Maoa* gene was also included in this filtered subset.

Then, we identified weighted co-expression network modules in which these genes are coordinately expressed with others. Since these modules did not significantly correlate with clinical traits across the entire cohort, we analyzed them separately for the ASD models and WT animals in each dataset. Quantitative topology analysis confirmed a consistent decrease in clustering coefficients in ASD models across both datasets, signaling a loss of functional coordination (Table 2).

However, the nature of this disorganization was model-specific. In the GSE144046 dataset (*Cul3* haploinsufficient mice), the ASD model showed a near-total collapse of connectivity and density. The genes in the module effectively ceased interacting, leading to a loss of structural integrity. In contrast, in the GSE144277 dataset (*Mbd5* haploinsufficiency), the module in the ASD model was twice as large (2100 vs. 980 genes) and demonstrated higher absolute connectivity. However, both density (0.55 in WT and 0.48 in the ASD model) and the clustering coefficient (0.64 in WT to 0.59 in the ASD model) decreased, confirming that while the network expanded massively, it became noisier and less efficient. This interpretation is further supported by the scaled connectivity, which showed a consistent decline in the ASD models, confirming that per-gene interaction strength is reduced regardless of whether the total module size expands or shrinks (Table 2).

We selected the genes that interact with *Comt* in the networks for further analysis (for GSE144046, *Maoa*-interacting genes also were included). GO BP enrichment analysis revealed similar trends across datasets. First of all, the significant number of genes that are co-expressed with genes of interest in WT still remains co-expressed with these genes in the ASD model.

**Figure 6:**
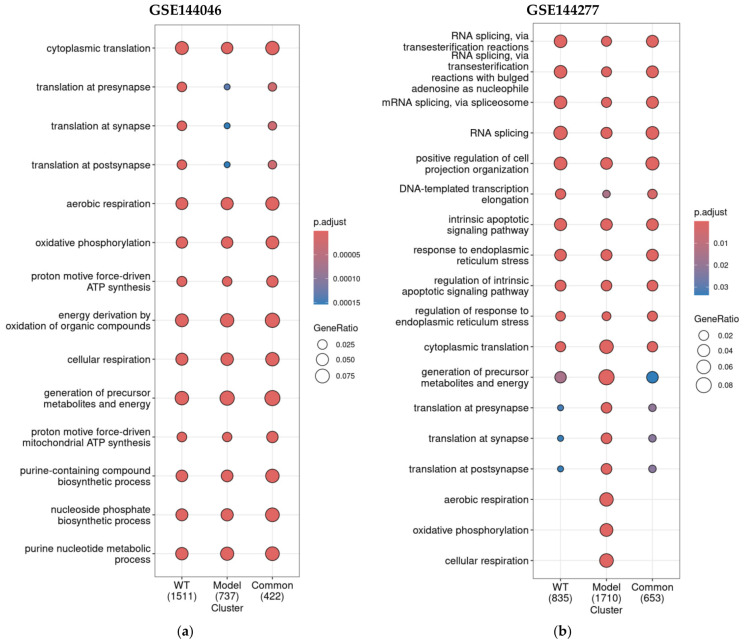
GO BP enrichment analysis results in genes co-expressed with *Comt* in WT mice and mouse ASD models. (**a**) WT and *Cul3* haploinsufficient mice (GSE144046); (**b**) WT and *MBD5*^+/GT^ mice (GSE144277). Dotplot presenting the enrichment of Gene Ontology Biological Process (GO BP) terms for the cluster of genes co-expressed with MAOB mRNA. The *y*-axis displays the enriched GO terms, while the *x*-axis represents the Gene Ratio. The size of the dots corresponds to the count of genes enriched in each pathway, and the color intensity indicates the statistical significance (adjusted *p*-value).

In both datasets, *Comt* was co-expressed with genes involved in translation, respiration, and synapse-specific translational processes. Notably, the impact of ASD modeling on these associations was contrasting. The *MBD5*^+/GT^ genotype was associated with a partial loss of these co-expression patterns (Figure 6a), whereas in *Cul3* haploinsufficient mice, the knockout led to more pronounced co-expression of *Comt* with synaptic translation genes compared with WT littermates (Figure 6b). Another discrepancy which was revealed in genes was their involvement in cellular respiration.

Values are constant between the two genotypes in GSE144046, but reach statistical significance only in ASD model in GSE144277.

### 2.6. Dopaminergic Genes Expression in the Cerebellum in Rat DAT-KO ADHD/ASD Model

**Figure 7:**
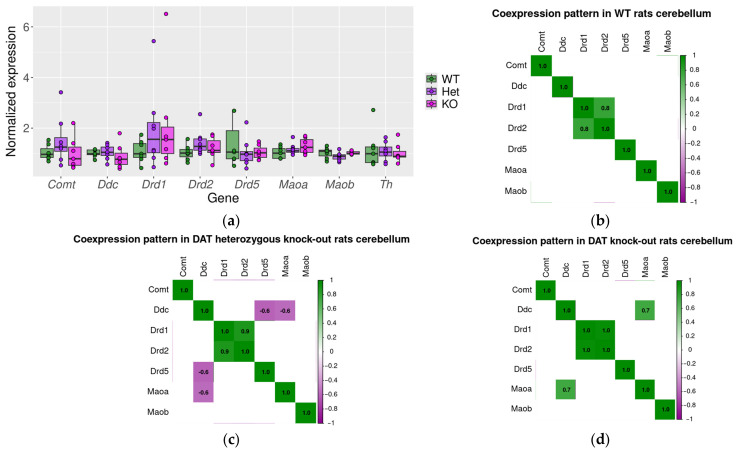
Dopaminergic gene expression (**a**); expression levels are represented as 2^−ΔΔCt^- normalized values; and co-expression (**b**–**d**) profiles in DAT-KO rats (RT-PCR data). The values indicate Spearman’s correlation coefficients for Ct values with statistical significance set at *p* < 0.05.

The expression of dopamine receptors’ genes *Drd1*, *Drd2*, and *Drd5*, as well as *Th*, *Ddc*, *Comt*, *Maoa*, and *Maob*, was identified in cerebellar samples harvested from DAT-KO line rats by RT-PCR. The results for *Slc18a2*, *Drd3*, and *Drd4* were unstable and poorly reproducible. No significant differences in expression levels were revealed in WT rats, rats with DAT knockout in the heterozygous condition (DAT-Het), and homozygous DAT knockout rats (DAT-KO) (Figure 7a).

In the same line with the findings detailed in the previous sections, within the DAT-KO and DAT-Het rats, the dopaminergic co-expression network demonstrates an increase in complexity, characterized by the emergence of novel interactions that were entirely absent in the WT group. Most intriguingly, the network topology appears to reach its highest level of structural complexity within the DAT-Het animals (Figure 7b–d).

## 3. Discussion

The major source of cerebellar dopamine originates from the outside structures, including the VTA and substantia nigra, and is represented by midbrain projections [17]. These structures’ functions are disorganized in ASD and ADHD [57,58,59], so it is reasonable to suggest that disruptions in this primary dopaminergic input might be reflected in the cerebellum at the molecular level. Therefore, it is plausible to expect alterations in the expression of dopaminergic genes within the cerebellum as a potential response to the dysregulation of this circuit. Identifying these molecular shifts is critical even if they represent secondary ‘passenger’ effects rather than primary drivers of the disorder.

Despite *TH*, *DDC*, *SLC18A2*, *SLC6A3*, and other dopaminergic gene expression in the cerebellum, their expression is not coordinated in this structure, as it was previously revealed in the substantia nigra [60]. The identified co-expression relationships between genes involved in dopamine synthesis, storage, signaling, and degradation were sporadic and failed to demonstrate reproducibility across independent datasets or our DAT-KO rats study in vivo. This discrepancy highlights a fundamental tissue-specific feature. While genes in a primary dopamine-producing structure operate as a centralized functional unit, their coordination is lost in a target region where dopamine serves a more modulatory role. Notably, more complex patterns were identified in whole-cerebellar mouse and rat samples than in fragments of human cerebellum. This suggests that co-expression might be regulated at the level of distinct cerebellar substructures, where the expression sites of these genes are spatially segregated, potentially masking correlations when analyzed in the narrow part of the cerebellum.

WGCNA is a well-established and robust framework for identifying biologically meaningful modules and uncovering hidden regulatory patterns within high-dimensional transcriptomic data [61,62]. So we applied this approach for comparative analysis. As in the case of dopaminergic gene co-expression described above, the results acquired in mouse and human data were discrepant. *MAOB* was the only gene successfully integrated into the co-expression network in GTEx human cerebellar hemisphere samples. This gene encodes monoamine oxidase B, which is primarily expressed by astrocytes. Due to the lack of DAT in astrocytes and the intracellular localization of MAOB, its contribution to dopamine degradation is limited by low intracellular dopamine availability [63].

While MAOB inhibitors are commonly used to support dopaminergic neurons in Parkinson’s disease, their neuroprotective effects are often attributed to indirect mechanisms rather than the direct protection of the dopamine pool from MAOB-dependent degradation [63,64]. Additionally, it has been proposed that MAOB activity may normalize dopamine tone in the striatum of psychotic patients [65]. Previous studies demonstrated *MAOB* expression correlation with neuroinflammatory and neurodegenerative conditions as well as glial activation [66,67]. Our WGCNA results are consistent with these multifaceted roles of *MAOB*, particularly its association with glial-mediated immune responses.

Analysis of the expression patterns of dopamine signaling-associated genes in the mouse cerebellum revealed several key trends both in neurodevelopmental disorders models and their WT littermates. First, across both datasets, the expression of these genes remained relatively stable (as evidenced by the selection of variable genes for WGCNA). However, *Comt* expression variability reaches the established level and allows the identification that the *Comt* gene is integrated in the same network with genes whose products are involved in transcription, translation, cell respiration, and synaptic translation. While acknowledging that broad network scales can capture non-specific basic cellular processes, the identified synaptic translation pathways were consistently shared across conditions and datasets. Given the tissue-specific role of this process, these findings point to a specific molecular signature rather than generic co-variance, though further validation remains warranted. Synaptic translation is essential for rapid neuronal tuning and plasticity [68,69], and is suggested to be dysregulated in ASD [70,71]. This pattern in general was reproducible in both datasets and different genotypes. Meanwhile, in both datasets, it was disintegrated in neurodevelopmental disorders models compared to their WT littermates; however, the mechanisms of this disintegration were principally distinct, which mirrors complex and multidirectional neurodevelopmental disorders pathogenesis.

The intriguing results in the present analysis are the opposite effects of the two studied models on this association. *Mbd5* haploinsufficient mice, commonly named *MBD5*^+/GT^, are the model for the rare human disease 2q23.1 deletion syndrome. This disease is associated with severe intellectual disability, seizures, sleep disturbances, and autistic-like behaviors. Model animals demonstrate the relevant phenotype with social behavior impairment, stereotypy, affected balance, and movement coordination [56,72]. In contrast, *Cul3* haploinsufficient mice demonstrate social and cognitive deficits, accompanied by hyperactive behavior. Additionally, one of the morphological features of this model is decreased cerebellum size [55]. Given the distinct motor phenotypes observed in these models, differences in cerebellar molecular alterations were to be expected, and the study of several additional models of ASD and ADHD could elucidate the relations of different phenotype modules on the identified expression network disturbances.

The interpretations of the present study’s findings must be weighed against several critical methodological constraints. Primary among these is the limited scope of data acquisition, as a search of the GEO repository yielded only two transcriptomic datasets that strictly adhered to our inclusion criteria. Consequently, our network module detection was constrained by these small-scale study groups, which limited statistical power and generalizability. We acknowledge that a methodological limitation of this study is the sample size used for the separate co-expression analyses (n = 6 for GSE144046 and n = 8 to 10 for GSE144277 subgroups), which falls below the original developer-recommended threshold of 15–20 samples for optimal global module detection. To mitigate the inherent sensitivity of smaller datasets to noise and outliers, network reconstruction was performed using the bioNERO pipeline [73], which implements data preprocessing and variance-stabilizing transformations to eliminate low-variance transcripts prior to network construction.

Another methodological limitation is related to the heterogeneous nature of the selected data. The GTEx data were included in the analysis, but they are not complementary to the mouse or rat samples, not only because of species-specific features of the dopamine system but more likely because the rodent cerebellum commonly is sequenced totally, which is technically impossible for the large human cerebellum. This anatomical heterogeneity highlights the limitations of treating whole-tissue rodent data as a direct proxy for human cerebellar structures in dopaminergic signaling studies and needs to be considered in meta-analyses and in cross-species translational model studies. The scarcity of available high-throughput data was compounded by the inherent biological complexity and phenotypic diversity of the neurodevelopmental models. For instance, while *Cullin3* haploinsufficiency is related to ASD-like behavior with hyperactivity and development anomalies, as well as DAT-KO, and *Mbd5* haploinsufficiency is not associated with hyperactive motor behavior, potentially introducing confounding variables into our results. Consequently, the divergent network responses observed between these models must be interpreted with caution. However, given their distinct genetic constructs, underlying molecular mechanisms, and phenotypes, these findings may highlight the specific pathway alterations unique to each model rather than demonstrating a universal systems-level phenomenon across different genetic backgrounds. Furthermore, because the analyzed datasets originated from small-scale study groups across diverse laboratory settings, our network findings should be interpreted with caution and warrant further validation in larger cohorts.

Despite all limitations described above, we revealed some common and reproducible trends in our analysis. First of all, despite the sample source, genes involved in dopamine signaling do not demonstrate pronounced co-expression. A little more apparent co-expression in different ASD/ADHD models needs further studies. The most expressed genes are *Comt* and *Mao*, which are involved in dopamine metabolism and utilization. These genes, particularly *Comt*, participate in co-expression networks with other genes that are linked to basic metabolic processes and synaptic translation, which is neuron-specific and principal for the development of ASD; however, these relationships tend to become disorganized in neurodevelopmental disorders models.

## 4. Materials and Methods

### 4.1. Gene Expression Omnibus Database Search

The RNAseq data were mined from the public database GEO from NCBI [74] by the search request (“cerebellum”[MeSH Terms] OR cerebellum[All Fields]) AND (ASD[All Fields] OR (“attention deficit disorder with hyperactivity”[MeSH Terms] OR ADHD[All Fields]) OR (“autistic disorder”[MeSH Terms] OR autism[All Fields]) OR (“autistic disorder”[MeSH Terms] OR autistic[All Fields]) OR hyperactivity[All Fields] OR (“attention”[MeSH Terms] OR attention[All Fields])) AND (“gsm”[Filter] AND “Homo sapience”[Organism] OR “Rattus norvegicus”[Organism] OR “Mus musculus”[Organism] AND “Expression profiling by high throughput sequencing”[Filter] AND (“10”[n_samples]: “10000”[n_samples])) in the GEO DataSets database. The inclusion criteria were as follows: (1) at least 5 cerebellar samples per study subgroup in the dataset, including intact WT samples; (2) the datasets represent the expression profiles of native samples; data for single-cell RNA sequencing or cell fractions were excluded.

Additionally, we included RNA-seq data for the neurotypical human cerebellum from the GTEx repository. This encompasses two distinct datasets: ‘Cerebellum’ and ‘Cerebellar Hemisphere’. These regions represent different sampling protocols; while the former consists of vermis tissue fixed in PAXgene (Qiagen, Hilden, Germany) at the donor site, the latter comprises fresh-frozen lateral hemisphere samples from the Miami Brain Bank [75]. Since methodological discrepancies—including fixative type and post-mortem intervals—can confound differential expression analysis, we processed and analyzed these datasets separately.

The data were downloaded from the GTEx portal. To ensure high transcriptomic quality, we selected only donors with a short clinical agony phase (Hardy scale 1 or 2). Furthermore, we filtered the datasets to include only genes with a minimum expression level exceeding 0.1 TPM in at least 50% of the samples.

### 4.2. Data Analysis

GTEx data were downloaded in TPM-normalized format. In contrast, data from GEO were obtained as raw counts, which were used for differential expression analysis (DEA) and subsequently CPM-normalized for correlation and weighted gene co-expression network analysis (WGCNA).

DEG was performed using the quasi-likelihood (QL) method in edgeR (v. 4.6.2) [76], following preliminary filtering with the filterByExpr function (default parameters). Data normalization was conducted using the calcNormFactors function, applying the trimmed mean of M-values (TMM) method. *p*-values were adjusted using the Benjamini–Hochberg procedure; genes with an adjusted *p*-value (FDR) < 0.05 were considered differentially expressed.

Co-expression modules were identified using the bioNERO Bioconductor package (v. 1.16.0) [73]. This tool was applied with default settings to identify dopamine signaling-associated genes within the networks and to select genes co-expressed with them across different conditions. After removing low-variance genes and outlying samples, the 5000 most variable genes were included in the analysis. Finally, Gene Ontology (GO) term enrichment analysis was performed on the identified clusters using the clusterProfiler package (v.4.6.0) [77].

All computational procedures are detailed in Supplementary Data S1–S3 for the GTEx, GSE144046, and GSE144277 datasets, respectively.

### 4.3. Animals

Cerebellum samples from 22 five-month-old adult male rats were harvested for the study. The experimental cohort comprised three genotype-specific groups, i.e., DAT knockout (DAT-KO, n = 7), heterozygous (DAT-Het, n = 8), and wild-type (WT, n = 7). All animals were obtained using a HET–HET breeding scheme, with their respective genotypes confirmed via a previously established protocol [46].

The research was conducted in strict accordance with the ethical guidelines approved by the Ethics Committee for Animal Research of Saint Petersburg State University (Protocol No. 131-03-2, 28 February 2025). During the study, rats were housed in an individually ventilated cage (IVC) system (RAIR IsoSystem World Cage 500; Lab Products, Inc., Seaford, DE, USA) under controlled environmental conditions, including a 12-h light/dark cycle (lights on at 9:00 a.m.), a temperature of 22 ± 1.0 °C, and relative humidity of 50–70%. Ad libitum access to standardized food and water was provided. Following the experimental period, rats were anesthetized with isoflurane and euthanized by decapitation for subsequent tissue harvesting.

### 4.4. RNA Isolation, Reverse Transcription, and Quantitative Polymerase Chain Reaction (qPCR)

Total RNA was extracted from tissue samples using ExtractRNA reagent (Evrogen, Moscow, Russia) following the manufacturer’s protocol. RNA concentration and purity were determined via spectrophotometry using a NanoDrop 2000 (Thermo Scientific, Waltham, MA, USA). For cDNA synthesis, 300 ng of total RNA from each sample was reverse-transcribed using the MMLV RT kit (Evrogen, Moscow, Russia) according to the manufacturer’s instructions. To monitor for potential genomic DNA contamination, No-Reverse Transcriptase (NRT) controls were included for each sample, and the absence of residual DNA was verified by the lack of amplification in these controls.

Quantitative PCR (qPCR) was employed to assess the relative levels of specific mRNAs using the CFX96 Touch Real-Time PCR Detection System (Bio-Rad, Hercules, CA, USA). The specific primer sequences utilized in this study are detailed in Table 3. All samples were analyzed at least in duplicate to ensure technical reproducibility.

The mRNA expression levels of *Th*, *Ddc*, *Drd1–5*, *Slc18a2*, *Comt*, *Maoa*, and *Maob* genes were quantified via qPCR using qPCRmix-HS SYBR (Evrogen, Moscow, Russia). To ensure primer specificity and the absence of non-specific products, a melting curve analysis was performed following the amplification cycles. Relative gene expression was determined using the 2^−ΔΔCt^ method, with the *Hprt* gene serving as the internal reference (housekeeping gene). The target gene expression in each sample was first normalized to *Hprt* (ΔCt) and subsequently compared to the mean of the wild-type (WT) control group (ΔΔCt), and results are expressed as 2^−ΔΔCt^.

### 4.5. Statistical Analysis

Data normality was verified using the Shapiro-Wilk test. Statistical significance was subsequently determined via one-way ANOVA followed by Tukey’s HSD post hoc test for normally distributed data (*p* > 0.05 for all groups). In cases where normality assumptions were not met, the Kruskal–Wallis test, followed by Mann-Whitney U post hoc analysis, was employed. Results were visualized using the ggplot2 R package (v. 3.5.2) [78].

Spearman’s rank correlation coefficients were calculated and visualized with the corrplot R package (v. 0.95) [79]. Correlations and differences were considered statistically significant at *p* < 0.05. Detailed statistical outputs are provided in Supplementary Data S4.

## 5. Conclusions

Almost all genes involved in dopamine synthesis, signaling, and metabolism are expressed in cerebellar samples, at least in whole cerebellum samples of laboratory mice. However, their expression seems to be less coordinated with one another in wild-type mice, rats, and humans without neurodevelopmental pathologies. There is a trend toward an increase in the co-expression of these genes in ASD or ASD/ADHD animal models, including *MBD5*^+/*GT*^ or *Cul3*^−/+^ mice or DAT-KO rats. However, it is weak and not reproducible between models. WGCNA allows us to identify a gene co-expression network involving the Comt gene, which is reproducible in two independent datasets, which represent two fundamentally different neurodevelopmental disorders models in mice. In this network, *Comt* is closely connected with genes involved in cell respiration, transcription, and translation. The identified network is deregulated in neurodevelopmental disorders mouse models; however, the pattern of the disorganization is background-dependent. In *MBD5*^+/*GT*^ mice, co-expression patterns seem to be destroyed, including the identified association between *Comt* and genes involved in synaptic translation regulation. At the same time, in *Cul3*^−/+^ mice, the network disorganization is accompanied by gene number expansion. The same applies to the number of genes co-expressed with *Comt* and involved in synaptic translation. However, this expansion weakens the network connectivity. The association of these two damaging effects with specific molecular or phenotypical features of two studied models needs further studies. Understanding the complex effects of ASD-related models on dopamine metabolism-related gene expression in the cerebellum may clarify new ways for more personalized medication for this spectrum condition.

## Figures and Tables

**Table 1:** RNAseq datasets included in the analysis.

GEO ID	Title	Platform	Groups and Model
GSE144046	Autism-linked Cullin3 germline haploinsufficiency severely impacts mouse brain development and cortical neurogenesis through RhoA signaling	Illumina HiSeq 4000 (Illumina, Inc., San Diego, CA, USA).	E3-ubiquitin ligase *Cullin3* (*Cul3*) mutations are associated with ASD. Its haploinsufficiency in mice is associated with hyperactivity, impaired memory, and social behavior [55]. 6 WT and 6 *Cul3*^−/+^ mice (P35, male and female in both groups).
GSE144277	Functional genomic consequences of MBD5 knockdown in mouse brain and CRISPR-derived neurons [mouse]	Illumina HiSeq 2000 (Illumina, Inc., San Diego, CA, USA).	*MBD5*, encoding the methyl-CpG-binding domain 5, is implicated in ASD, and its heterozygous knockout in mice (*MBD5*^+/GT^) leads to abnormal social behavior, cognitive impairment, craniofacial abnormalities, and motor deficits [56]. 10 *MBD5*^+/GT^ mice, 8 WT mice (8 weeks old, male and female in both groups).

**Table 2:** Network topology metrics for the identified *Comt*-containing modules.

Dataset	GSE144046		GSE144277	
Group	WT	Model	WT	Model
Gene count	1775	1093	980	2100
Density	0.17	0.07	0.55	0.48
Clustering coefficient	0.34	0.23	0.64	0.59
Mean connectivity	313.7	74.8	536.4	1012.8
Scaled connectivity	0.18	0.068	0.548	0.482

**Table 3:** Primers and probes were used for quantitative RT-PCR in DAT-KO rat study.

Target	Gene Name	Forward Primer	Reverse Primer
*Th*	Tyrosine hydroxylase	CGCTTCTTGAAGGAGCGGACTG	GCATGGCGGATATACTGGGTGC
*Ddc*	DOPA-decarboxylase	TGGCGTGGAGTTTGCAGATTCC	GTCCTGGTGACTGTGCCTCAGA
*Drd1*	Dopamine receptor D1	CGACACCTGAGGTCCAAGGTGA	CGCTGATCACGCAGAGGTTCAG
*Drd2*	Dopamine receptor D1	CTTGAAGAGCCGTGCCACCC	TGTCTGCCTTCCCTTCTGACCC
*Drd3*	Dopamine receptor D1	ACAGGTACACAGCGGTGGTCAT	GCAGGTGTGACAAAAGGGGGTC
*Drd4*	Dopamine receptor D1	GGACAGGTTTGTGGCTGTGACC	TCAGGAAGGCCCCAACTACCAC
*Drd5*	Dopamine receptor D1	CGGAGAACTGTGACTCCAGCCT	CTTCTTGATGGACGCTCGCAGG
*Slc18a2*	Solute carrier family18 member A2	TGTCTGCCTTCCCTTCTGACCC	AGGAGTCCACCATCCCAATTGCA
*Comt*	Catechol-O-methyltransferase	AACCCTGACTACGCTGCCATCA	AGCAGGCCACATTTCTCCAGGA
*Maoa*	Monoamineoxidase A	GTCCAAGGATGTTCCAGCCA	ATCTTGAGCAGACCAGGCAC
*Maob*	Monoamineoxidase B	AACTGCGGAGACCCATGAGGTT	GGCCTCTCCAGCTTCACTCTGT
*Hprt* ^1^	Hypoxanthine phosphoribosyltransferase1	ATGGACTGATTATGGACAGGAC	GCAGGTCAGCAAAGAACTTATAGCC

^1^ Applied as the reference gene for results normalization.

## Data Availability

The datasets used for secondary analysis are available via the GTEx portal and the Gene Expression Omnibus (GEO) database. The code used for the analysis is provided in the Supplementary Materials.

## Supplementary Materials

The following supporting information can be downloaded at: https://rpubs.com/aeTerner/Supp_1_cer_2026 (accessed on 30 April 2026)—GTEx data analysis, https://rpubs.com/aeTerner/Supp_2_cer_2026 (accessed on 30 April 2026)—GSE144046 data analysis, https://rpubs.com/aeTerner/Supp_3_cer_2026 (accessed on 30 April 2026)—GSE144277 data analysis, https://rpubs.com/aeTerner/Supp_4_cer_2026 (accessed on 30 April 2026)—qRT-PCT data analysis.

## Abbreviations

The following abbreviations are used in this manuscript:

ADHD	Attention deficit/hyperactivity disorder
ASD	Autistic spectrum disorders
cDNA	Coding DNA
COMT	Catechol-O-methyltransferase
DAT	Dopamine transporter
DEG	Differently expressed genes
GEO	Gene Expression Omnibus
GTEx	Genotype-Tissue Expression
Het	Heterozygote
KO	Knockout
MAO	Monoamine oxidase
PCs	Purkinje cells
qPCR	Quantitative polymerase chain reaction
RT	Reverse transcription
TPM	Transcripts per million
VTA	Ventral tegmental area
WGCNA	Weighted gene co-expression network analysis
WT	Wild type
